# Enzyme-Based Antiviral Potential of *Cinnamomum verum* J. Presl. Essential Oil and Its Major Component (*E*)-Cinnamaldehyde

**DOI:** 10.1021/acsomega.3c09595

**Published:** 2024-03-13

**Authors:** Ayşe Esra Karadağ, Sevde Nur Biltekin, Usman Ghani, Betül Demirci, Fatih Demirci

**Affiliations:** † Department of Pharmacognosy, School of Pharmacy, Istanbul Medipol University, 34810 Beykoz, Istanbul, Turkey; ‡ Department of Pharmaceutical Microbiology, School of Pharmacy, Istanbul Medipol University, 34810 Beykoz, Istanbul, Turkey; § Department of Pathology, Clinical Biochemistry Unit, College of Medicine, King Saud University, Riyadh 12372, Saudi Arabia; ∥ Department of Pharmacognosy, Faculty of Pharmacy, Anadolu University, 26470 Eskişehir, Türkiye; ⊥ Faculty of Pharmacy, Eastern Mediterranean University, Mersin 10, 99450 Famagusta, North Cyprus, Türkiye

## Abstract

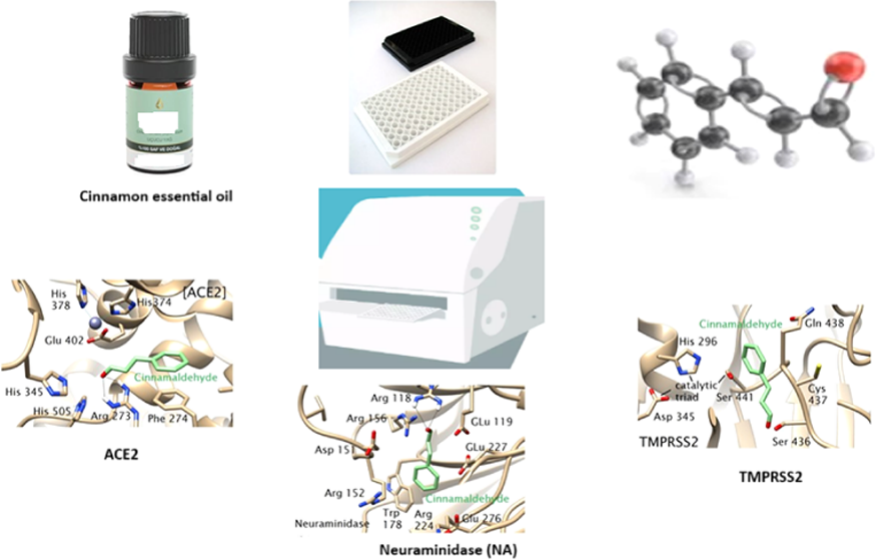

In the present study, *Cinnamomum verum* J. Presl. bark essential oil and its main component cinnamaldehyde was evaluated *in vitro* for neuraminidase (NA), transmembrane serine protease (TMPRSS2), and angiotensin converting enzyme 2 (ACE2) inhibitory activities. The chemical composition of *C. verum* essential oil was confirmed by both gas chromatography–mass spectrometry (GC/MS), and gas chromatography–flame ionization detection (GC-FID), where 75.9% (*E*)-cinnamaldehyde was the major component. The ACE2, NA, and TMPRSS2 enzyme inhibitions of *C. verum* bark essential oil at 20 μg/mL concentration, and (*E*)-cinnamaldehyde (5 μg/mL) were calculated and compared in the range of 54.2–89.9%. Molecular docking results supported that (*E*)-cinnam-aldehyde was specific to ACE2 with 89.9% inhibition. Our findings suggest further *in vivo* studies to confirm the effective and safe use of the essential oil as well as the (*E*)-cinnamaldehyde.

## 1 Introduction

*Cinnamomum verum* J. Presl. (Lauraceae) essential oil is generally used for its flavor and fragrance in different food and fragrance industries. Its major component is an aromatic aldehyde, cinnamaldehyde. In addition to its culinary uses, *C. verum* bark preparations are known to be used ethnobotanically for the treatment of viral diseases, especially flu and colds. It is also used against indigestion problems.^1^ The industrial product *C. verum* essential oil and extracts are of high value to the cosmetic and food industry due to its characteristic aroma. However, it is also known to be commercially adulterated with *Cinnamomum cassia*. Due to the relatively high levels of coumarin that *C. cassia* contains, it is not recommended to use it for a long time and in large amounts. *C. verum* is safer to use since it contains relatively lower amounts of coumarin derivatives. *C. verum* essential oil is used for its antimicrobial and larvicidal effects.^2^ Previous studies using *C. verum* essential oil showed remarkable antimicrobial activity against various human resistant pathogens.^3^ Cinnamaldehyde has also been tested in pure form against different pathogens where it showed as relative efficacy.^4^ The ethanol extract of *C. verum* ethanol was also previously evaluated *in vitro* against SARS-CoV-2.^5^ Moreover, recent research on *C. verum* water and ethanol extracts were reported to inhibit the binding of SARS-CoV-2 spike protein to ACE2 in a dose-dependent manner.^6^

Essential oils are known for their antiviral activity against human herpes viruses, HIV, and influenza viruses.^7^ Volatile components of essential oils have lipophilic properties that are helpful in disrupting viral membrane integrity with the potential to penetrate the membrane.^8^ They also disrupt viral replication, benefiting the host respiratory system through mucus lysis and bronchodilation.^9^

Influenza viruses including SARS-CoV-2 that causes COVID-19, utilize spike protein cleavage for ACE2 cell-surface receptor recognition and entry into the host cells.^10^ The spike protein is proteolytically cleaved by transmembrane protease serine 2 (TMPRSS2), which belongs to trypsin-like serine proteases. Its function also includes enzymatic remodeling of the extracellular matrix. Unregulated activities of TMPRSS2 and related proteases are also known to be responsible for the pathogenesis of cancer and metastasis. The enzymes are an important target for the treatment of cancer and disabling influenza viral entry into the host cells.^11^

The present work evaluates the *in vitro* antiviral and enzyme inhibitory effects of *C. verum* essential oil and its major compound cinnamaldehyde targeting ACE2, neuraminidase, and RMPRSS2 enzymes. It also involves the molecular docking of cinnamaldehyde with the target enzymes to determine its respective binding modes. To the best of our knowledge, this is the first study of the target enzyme inhibitory activities of *C. verum* essential oil and its major constituent cinnamaldehyde.

## 2 Results and Discussion

### 2.1 GC/MS and GC-FID Analyses

The essential oil composition of *C. verum* was verified and is listed in Table 1, where relative percentages of each constituent sum up with a total of 96%. The major constituent was characterized as (*E*)-cinnamaldehyde (75.9%). Other components of the essential oil included linalool (7%), O-methoxycinnamaldehyde (2.7%), α-copaene (2.3%), δ-cadinene (2.2%), and α-muurolene (1.8%), respectively. The analytical results of this present study fully complied with the European Pharmacopoeia in terms of its (*E*)-cinnamaldehyde, linalool, and O-methoxycinnamaldehyde contents.^12^

**Table 1:** *C. verum* Essential Oil Chemical Composition^a^

RRI	compound	%: relative percentages	IM
1087	butyl acetate	0.2	MS
1274	1,3,5-trimethylbenzene	1.0	MS
1280	*p*-cymene	0.1	*t*_R_, MS
1497	α-copaene	2.3	MS
1553	linalool	7.0	*t*_R_, MS
1612	β-caryophyllene	1.0	*t*_R_, MS
1687	α-humulene	0.1	*t*_R_, MS
1704	γ-muurolene	0.4	MS
1740	α-muurolene	1.8	MS
1773	δ-cadinene	2.2	MS
1776	γ-cadinene	0.1	MS
1853	calamenene	0.4	MS
2068	(*E*)-cinnamaldehyde	75.9	*t*_R_, MS
2209	T-muurolol	0.4	MS
2219	δ-cadinol	0.3	MS
2282	O-methoxycinnamaldehyde	2.7	MS
2308	cinnamyl alcohol	0.4	*t*_R_, MS
	total	96.3	

a RRI Relative retention indices calculated against *n*-alkanes. % calculated from FID data. IM: Identification method; *t*_R_, identification based on the retention times of genuine compounds on the HP Innowax column; MS, identified on the basis of computer matching of the mass spectra with those of the Wiley and MassFinder libraries and comparison with literature data.

The relative percentages of the other components of the essential oil were found to be variable compared to previous studies, however, our analytical results are comparable with that of previous studies on the cinnamaldehyde ratios found in *C. verum* essential oil.^4,13^

### 2.2 *In Vitro* Studies

The *C. verum* essential oil and (*E*)-cinnamaldehyde were tested at a concentration of 20 μg/mL and for 5 μg/mL enzyme inhibitory activity, respectively. The cinnamon essential oil showed 67% inhibition of ACE2, 54.2% of Neuraminidase, and 70.5% of TMPRSS2 (Table 2). (*E*)-cinnamaldehyde enzyme inhibition results were found as 89.9, 87.4, and 85.5% against the same enzymes. The results were also compared with positive controls using commercial kits.

**Table 2:** Enzyme Inhibitory Activity of *C. verum* Essential Oil and (*E*)-cinnamaldehyde

	inhibition (%)		
target enzyme	essential oil	(*E*)-cinnamaldehyde	positive control^a^
ACE2	67.02 ± 0.43	89.91 ± 1.12	98.4 ± 0.1
neuraminidase	54.18 ± 1.48	87.42 ± 0.91	99.1 ± 0.21
TMPRSS2	70.49 ± 1.20	85.51 ± 0.7	96.7 ± 0.17

a Provided within the commercial kits.

Our work showed that *C. verum* bark essential oil is more effective on TMPRSS2 and ACE2 enzymes than on neuraminidase. Since TMPRSS2 and ACE2 are important enzymes for influenza viral entry into the host cells, it is highly desirable to investigate the direct effects of the essential on influenza viruses including coronavirus. Interestingly, the ethnobotanical use of *C. verum* bark also supports the findings,^1^ including inhibition of ACE2 by the polar extract of *C. verum*(6) We also conducted molecular docking of cinnamaldehyde, the major component of the essential oil, with the target enzymes for insights into its binding mode for each enzyme. In the *in vitro* assay, unlike essential oil, cinnamaldehyde has results that inhibited more ACE2 enzymes. Although the enzyme inhibition results of cinnamaldehyde are generally similar to those of essential oil, they are more consistent with molecular docking studies. However, in general, it was observed that cinnamaldehyde inhibits ACE2 and NA enzymes more.

The essential oil *C. verum* is known to exhibit antimicrobial activity against human pathogens^14^ and anti-HIV activity.^14^ However, to date, no study has been conducted on the anti-influenza viral effects of the essential oil. This is the first work on the *C. verum* essential oil that focuses on the control of influenza viruses through inhibition of ACE2, TMPRSS2, and neuraminidase enzymes.

### 2.3 Molecular Docking of Cinnamaldehyde

#### 2.3.1 ACE2-Cinnamaldehyde Binding Mode

Each binding pose was inspected for binding score and molecular interactions that included hydrogen bonding, hydrophobic contacts, and other interactions. Table 3 lists the binding energies of cinnamaldehyde for the target enzymes.

**Table 3:** Binding Energies of Cinnamaldehyde for the Target Enzymes

enzyme	cinnamaldehyde binding energy (kcal/mol)
ACE2	–5.50
neuraminidase	–5.40
TMPRSS2	–4.30

ACE2 carboxypeptidase contains two subdomains: I and II. The zinc-containing subdomain I is made of amino acids starting from 19 to 20, 290 to 397, and 417 to 430 residues, whereas the subdomain II is formed of 103–289, 398–416, and 431–615 residues.^15^ Both of the subdomains form the active site of the enzyme. The catalytic center is composed of S1 and S1′ subsites for substrate or inhibitor binding. Docking results showed that it is the S1 site in the subdomain II to which cinnamaldehyde interacted where it crucially established two hydrogen bonds with the side chain of Arg 273 through its carbonyl oxygen.

**Figure 1:**
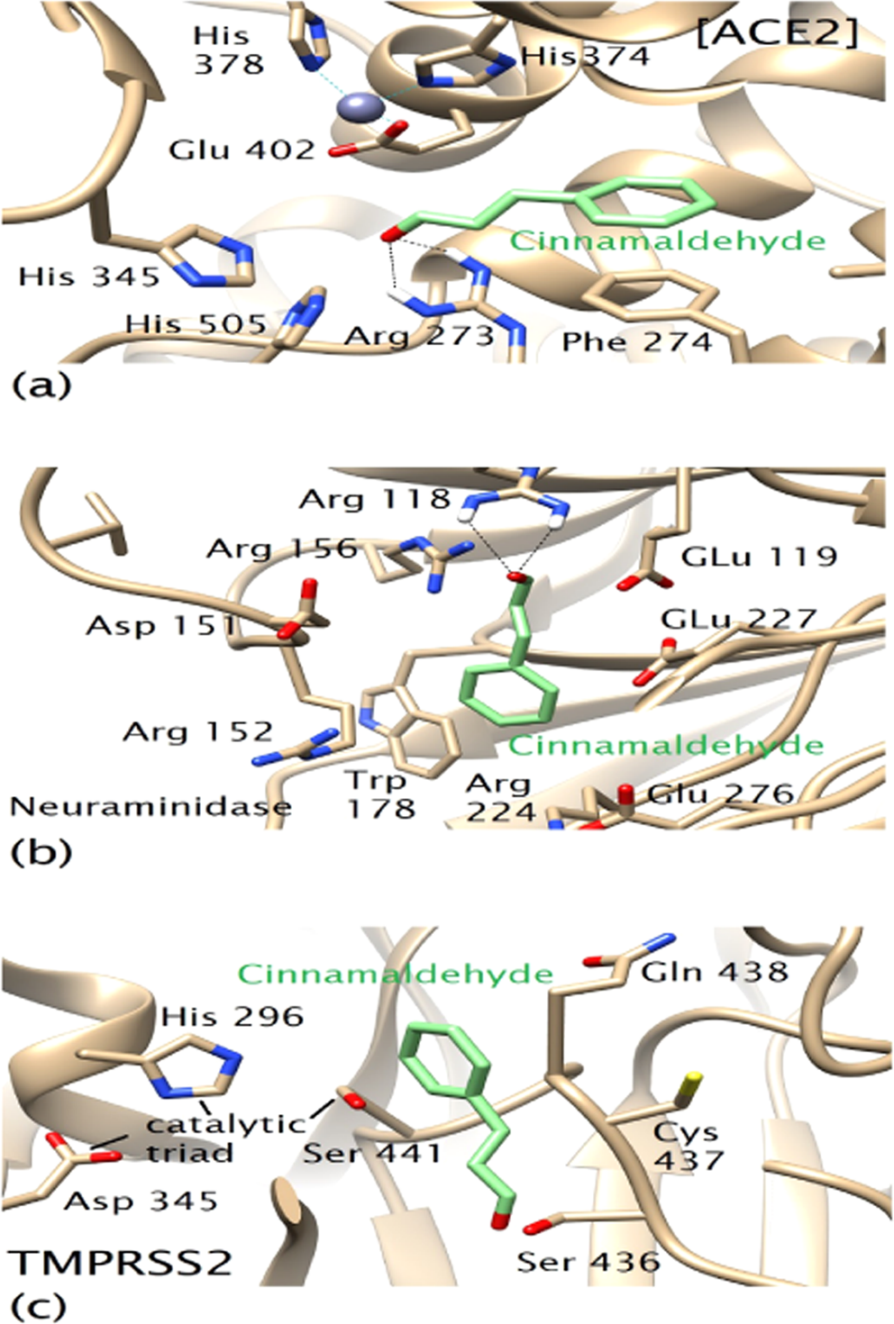
(a) Cinnamaldehyde prefers to bind in the S1 site of ACE2 subdomain II. The zinc center located in subdomain I can be seen in the background. Black dotted lines represent hydrogen bonds. (b) The binding mode of cinnamaldehyde for neuraminidase features substrate-like bidentate hydrogen bonding with Arg 118. To some extent, the binding also is similar to that of oseltamivir. (c) Hydrophobic interaction of cinnamaldehyde with Gln 438 in the S3 subsite of TMPRSS2 away from the catalytic triad: Ser 441, His 296, and Asp 345.

The compound was otherwise stabilized by hydrophobic contacts and π–π-stacking of its phenyl ring with that of Phe 274 residue, as illustrated in Figure 1a. These were the two main molecular interactions that conferred stability to the whole structure of cinnamaldehyde in the ACE2 active site. The results suggested that cinnamaldehyde demonstrated drug-like binding in the active site compared to known ACE2 inhibitors involving common amino acid residues for molecular interactions.

#### 2.3.2 Neuraminidase-Cinnamaldehyde Binding Mode

Hemagglutinin and neuraminidase enzymes are the two glycoproteins that are present in influenza virus membranes. The former involves host cell-surface binding through sialic acid to facilitate viral infection.^16^ The latter cleaves sialic acid from the virus to mediate virus release and spread infection after cellular replication. Classification of influenza-A viruses is based on the types of hemagglutinin and neuraminidase that they contain (hemagglutinins H1–H16 and Neuraminidase N1–N9). Examples of these include H1N1, H2N2, and H3N2 viruses with a history of pandemics in the past. Neuraminidase is an important target for designing anti-influenza drugs that has led to the discovery of zanamivir and oseltamivir, which are fairly transition state analogs of the substrate. The active site of all influenza neuraminidases is identical featuring three arginine residues Arg 118, 292, and 371, which bind the carboxylate group of the sialic acid substrate in addition to other interactions. Neuraminidases from various influenza viruses contain a 150-loop composed of residues 147–152 and a 150-cavity adjacent to the active site. The crystal structure of N8 neuraminidase in complex with oseltamivir suggests that it forms a hydrogen bond with Tyr 347 via the C1 carboxylate group augmented by a bidentate hydrogen bond of the same group with Arg 371.^16^ Similar to oseltamivir, cinnamaldehyde also preferred to bind in the same active site region of N8 neuraminidase; however, it interacted with different residues. It involved Arg 118 for bidentate hydrogen bonding with its carboxylate group, which is similar to substrate binding. Interestingly, it also targeted Arg 152 of the 150-loop via hydrophobic interactions in addition to Trp 178 and Arg 224 (Figure 1b). To some extent, the binding mode of cinnamaldehyde features substrate-like interactions that also share some similarities to oseltamivir binding. Cinnamaldehyde can be a promising candidate for anti-influenza virus drug development either alone or as a hybrid product with other chemical scaffolds.

#### 2.3.3 TMPRSS2-Cinnamaldehyde Binding Mode

The active site of TMPRSS2 is highly conserved within all related serine proteases that matches with chymotrypsin and trypsin fold bearing a six-stranded β barrel leading to the catalytic triad of Ser 441, His 296, and Asp 345 amino acids. The active site subsites include S4–S3–S2–S1–S1′–S2′–S3′–S4′ that bind the P4–P3–P2–P1–P1′–P2′–P3′–P4′ substrate with the proteolytic cleavage site at P1–P1′. A number of TMPRSS2 inhibitors have been studied to determine the mechanism of inhibition of the enzyme.^11^ For comparative discussion, it is important to mention the crystal structure of nafamostat in complex with TMPRSS2.^11^ It is a potent inhibitor of the enzyme that binds in the S1 subsite, forming an acyl-enzyme complex with the catalytic Ser 441. The phenylguanidino group of the inhibitor settles in the S1 subsite where it forms salt bridges with the highly conserved Asp 435, Ser 436, and Gly 464 residues. The S2 subsite with conserved Lys 342 prefers small or electronegative substrates, whereas the S3 and S4 subsites containing Gln 438 and Thr 341, respectively, prefer a variety of amino acids. Our docking results showed that cinnamaldehyde preferred the S3 subsite where it hydrophobically interacted with Gln 438. It is oriented toward Gln 438 and away from the catalytic triad, therefore, it neither interacted with the catalytic Ser 441 nor formed a hydrogen bond with any residue in the active site, as in Figure 1c. In fact 2,4-dihydorxycinnamic acid is the final acyl-enzyme product of 7-hydorxycoumarin-chymotrypsin complex.^17^ In this regard, cinnamaldehyde mimics the acyl product of coumarins that react with serine proteases.

Overall, the binding modes of cinnamaldehyde for ACE2, neuraminidase, and TMPRSS2 provide clues to understanding the mechanism of inhibition of the enzymes and for designing new antiviral and potential anticancer drugs. According to the results, the hydroxyl group is important for establishing molecular contacts with the active site residues, especially with ACE2 and neuraminidase. Cinnamaldehyde may possess drug-like properties that can be further explored at higher levels of *in vivo* work.

## 3 Materials and Methods

The ACE2 enzyme inhibitor assay kit (K310) was obtained from BioVision, Waltham, MA. TMPRSS2 and Neuraminidase kits were purchased from Abcam (BPS Bioscience 78083, San Diego, CA) and Sigma-Aldrich (MAK121, Darmstadt, Germany), respectively. The *C. verum* bark essential oil was supplied by Doalinn (İstanbul, Türkiye). The voucher sample was deposited at the IMEF Herbarium (Herbarium No: IMEF 1191).

### 3.1 GC-FID and GC/MS Analyses

The GC-FID analysis was performed using the FID detector at 300 °C (Agilent 6890N GC system, CA). Simultaneous automatic injection was carried out by using the same conditions in two identical columns in the GC/MS system (Agilent 5975 GC-MSD). Relative percentages of the volatile components were calculated by using the FID chromatograms. This process was performed by the GC/MS MassFinder3 Library, and in-house Baser Library of Essential Oil Constituents by analyzing either authentic samples or the relative retention index (RRI) of *n*-alkanes.^18^

### 3.2 *In vitro* Enzyme Inhibitory Activity

The standard protocols of the enzyme assay kits were followed. Stock solutions of the test substances were prepared in DMSO (1%, *v/v*) and aliquots of essential oil (20 μg/mL) and (*E*)-cinnamaldehyde (5 μg/mL) were transferred to each well. The enzyme solution was added to all wells except the blank, followed by the addition of the substrate solution (40 μL) to each well. The enzyme reaction was measured in a SpectraMax i3 microplate reader (Molecular Devices, CA) in fluorescence mode after incubation for 30 min at 37 °C. The microplate reader was set at Ex/Em and VU/visible wavelengths specific for each enzyme assay according to the kit protocol. Camostat (BPS Bioscience, 78083, San Diego, CA) was used as a positive control for TMPRSS2 assays. Other positive control substances cannot be named because they are not clearly stated in the kit content.

The results were calculated as % inhibition values, which were retrieved from triplicate experiments.^19^

### 3.3 Molecular Docking

Molecular docking of (*E*)-cinnamaldehyde with all target enzymes was performed in AutoDock Vina (v. 1.2.0) embedded in UCSF Chimera 1.14, build 42094, (University of San Francisco).^20,21^ All crystal structures of the enzymes were obtained from the Protein Databank with accession codes as follows: human ACE2 (1R4L),^15^ influenza A virus neuraminidase (2HU0),^16^ and human TMPRSS2 (7MEQ).^11^

The proteins and ligands were prepared for docking in UCSF Chimera according to the protocol described in our previous work.^22^ Briefly, docking was performed in AutoDock Vina (v 1.2.0) that yielded multiple binding poses of cinnamaldehyde for each target enzyme. All of the top-ranked poses with low energy were found to be located within the active sites of the enzymes. Each selected pose was examined and compared with the crystal structures of respective enzymes in complex with known inhibitors. Verification of all molecular interactions of cinnamaldehyde was conducted using the PLIP server.^23^ UCSF Chimera was used to create figures for the binding poses.

### 3.4 Statistical Analysis

The statistical analysis was carried out using GraphPad Prism, v7.02 (GraphPad, La Jolla, CA). The data were expressed as the mean with standard deviation. A value of *p* < 0.05 was accepted as statistically significant.

## 4 Conclusions

Our work showed that cinnamon essential oils carry promising pharmacological potential other than aromatherapy applications. With its ability to inhibit ACE2, TMPRSS2, and neuraminidase *in vitro*, cinnamon essential oil and its constituents can be promising candidates for the development of drugs against influenza viruses. The binding modes of cinnamaldehyde for ACE2, neuraminidase, and TMPRSS2 provided clues to understanding the mechanism of inhibition of the enzymes and for designing new antiviral and anticancer drugs. Its binding modes share some similarities to that of known potent inhibitors of the target enzymes. Cinnamaldehyde may possess drug-like properties that can be further explored at higher levels of *in vivo* work.

## Dedication

The authors would like to dedicate this work to the memory of Prof. Dr. Erdem Yeşi̇lada who passed away on 27.7.2023.
